# Evaluating the usefulness of considering the size and morphological type of type B2 vessel area based on Japan Esophageal Society classification in estimating tumor invasion depth in superficial esophageal squamous cell carcinomas: study protocol for a prospective observational study (Japan BEES study)

**DOI:** 10.1186/s12876-024-03138-6

**Published:** 2024-01-26

**Authors:** Masao Yoshida, Keita Mori, Yuji Urabe, Dai Hirasawa, Fumisato Sasaki, Manabu Takeuchi, Tomohiro Kadota, Toshiyuki Yoshio, Shigetaka Yoshinaga, Yoko Kitamura, Kazuya Ohno, Yoichiro Ono, Kimihiro Igarashi, Hiroaki Takahashi, Ryu Ishihara

**Affiliations:** 1 Division of Endoscopy, Shizuoka Cancer Center, Shizuoka, Japan; 2 Clinical Research Center, Shizuoka Cancer Center, Shizuoka, Japan; 3 Gastrointestinal Endoscopy and Medicine, Hiroshima University Hospital, Hiroshima, Japan; 4 Department of Gastroenterology, Sendai Kousei Hospital, Miyagi, Japan; 5 Digestive and Lifestyle Diseases, Kagoshima University Graduate School of Medical and Dental Sciences, Kagoshima, Japan; 6 Division of Gastroenterology and Hepatology, Nagaoka Red Cross Hospital, Nagaoka, Japan; 7 Department of Gastroenterology and Endoscopy, National Cancer Center Hospital East, Chiba, Japan; 8 Department of Gastroenterology, Cancer Institute Hospital, Tokyo, Japan; 9 Endoscopy Division, National Cancer Center Hospital, Tokyo, Japan; 10 Department of Gastroenterology and Hepatology, Center for Digestive and Liver Diseases, Nara City Hospital, Nara, Japan; 11 Department of Gastroenterology, Shizuoka General Hospital, Shizuoka, Japan; 12 Department of Gastroenterology, Fukuoka University Chikushi Hospital, Fukuoka, Japan; 13 Department of Gastroenterology, Keiyukai Daini Hospital, Hokkaido, Japan; 14 Department of Gastrointestinal Oncology Osaka International Cancer Institute, Osaka, Japan

**Keywords:** Magnifying endoscopy, Endoscopic diagnosis, Japan Esophageal Society Classification, B2 vessel, B2 vessel area, Morphological type, Esophageal cancer, Squamous cell carcinoma

## Abstract

**Background:**

Accurate evaluation of tumor invasion depth is essential to determine the appropriate treatment strategy for patients with superficial esophageal cancer. The pretreatment tumor depth diagnosis currently relies on the magnifying endoscopic classification established by the Japan Esophageal Society (JES). However, the diagnostic accuracy of tumors involving the muscularis mucosa (MM) or those invading the upper third of the submucosal layer (SM1), which correspond to Type B2 vessels in the JES classification, remains insufficient. Previous retrospective studies have reported improved accuracy by considering additional findings, such as the size and macroscopic type of the Type B2 vessel area, in evaluating tumor invasion depth. Therefore, this study aimed to investigate whether incorporating the size and/or macroscopic type of the Type B2 vessel area improves the diagnostic accuracy of preoperative tumor invasion depth prediction based on the JES classification.

**Methods:**

This multicenter prospective observational study will include patients diagnosed with MM/SM1 esophageal squamous cell carcinoma based on the Type B2 vessels of the JES classification. The tumor invasion depth will be evaluated using both the standard JES classification (standard-depth evaluation) and the JES classification with additional findings (hypothetical-depth evaluation) for the same set of patients. Data from both endoscopic depth evaluations will be electronically collected and stored in a cloud-based database before endoscopic resection or esophagectomy. This study’s primary endpoint is accuracy, defined as the proportion of cases in which the preoperative depth diagnosis matched the histological depth diagnosis after resection. Outcomes of standard- and hypothetical-depth evaluation will be compared.

**Discussion:**

Collecting reliable prospective data on the JES classification, explicitly concerning the B2 vessel category, has the potential to provide valuable insights. Incorporating additional findings into the in-depth evaluation process may guide clinical decision-making and promote evidence-based medicine practices in managing superficial esophageal cancer.

**Trial registration:**

This trial was registered in the Clinical Trials Registry of the University Hospital Medical Information Network (UMIN-CTR) under the identifier UMIN000051145, registered on 23/5/2023.

## Background

Esophageal cancer is among the ten most common cancers; it has high morbidity and mortality. Following the National Comprehensive Cancer Network Guidelines, endoscopic resection (ER) is the preferred standard treatment for clinical T1a (cT1a) esophageal cancers without metastasis. However, esophagectomy or chemoradiotherapy (CRT) is recommended for clinical T1b (cT1b) esophageal cancers [1]. ER for esophageal cancers has been extensively developed in Japan and is widely used as a radical treatment. Based on a nationwide clinical survey in Japan, the 5-year overall survival (OS) rates were 88.5% for pathological T1a (pT1a) and 77.9% for pathological T1b (pT1b) cases treated with ER. In contrast, the 5-year OS rate was 83.6% for pT1a and 73.8% for pT1b cases treated with esophagectomy [2]. Therefore, based on these results, Japanese guidelines recommend ER for cT1a-epithelial/lamina propria (EP/LPM) cancers. In addition, ER is considered a viable option for cT1a-muscularis mucosa (MM) and cT1b-SM1, defined as tumors invading the upper third of the submucosal layer [3, 4]. However, for cT1b-SM2-3, esophagectomy or CRT remains the standard treatment. The efficacy of adding CRT after ER in pT1b cancers was confirmed in a non-randomized trial, which demonstrated a favorable 3-year OS rate of 90.7%. Consequently, ER with CRT is considered an alternative treatment for pT1b cancer [5]. Proper differentiation of EP/LPM, MM/SM1, and SM2-3 cancers before treatment is crucial for selecting the appropriate treatment.

Evaluating the preoperative tumor invasion depth using magnifying endoscopy enables the diagnosis of cancer invasion by considering morphological changes in the vessels. To assess tumor invasion depth, the Japan Esophageal Society (JES) proposed a magnifying endoscopic classification (Table 1) [6]. This classification is specialized to discriminate between cancer and non-cancer cases. It helps estimate the tumor invasion depth in cancer cases, specifically for the sub-classification of cEP/LPM, cMM/SM1, and cSM2-3. Type A vessels are diagnosed as non-cancerous, whereas Type B vessels are cancerous. Type B vessels are further classified into types B1, B2, and B3, corresponding to cEP/LPM, cMM/SM1, and cSM2-3. However, previous retrospective studies have revealed that the positive predictive value (PPV) of cEP/LPM as a Type B1 vessel was 92.4%, and that of cSM2 as a Type B3 vessel was 90.7%. However, the PPV of cMM/SM1 as a Type B2 vessel was only 55.7% (27.4% for overestimated pEP/LPM, 55.7% for accurate pMM/SM1, and 17.0% for underestimated pSM2 vessels). This indicates the need to improve the diagnostic accuracy of Type B2 vessels in the preoperative evaluation of tumor invasion depth.

**Table 1:** Japan Esophageal Society magnifying endoscopic classification and corresponding depth of invasion

Type of vessels	Definitions	Tumor invasion depth
Type A	Normal intrapapillary capillary loops or abnormal microvessels without severe irregularity	Non-cancerous
Type B	Abnormal microvessels with severe irregularity or highly dilated abnormal vessels	
B1	Type B vessels with a loop-like formation	EP/LPM
B2	Type B vessels without a loop-like formation	MM/SM1
B3	Highly dilated Type B vessels whose calibers appear to be more than thrice that of usual B2 vessels	SM2-3

*MM* Muscularis mucosa, *SM1* Upper third of the submucosal layer, *EP/LPM* Epithelial/lamina propria

Notably, several retrospective studies have been conducted to improve the diagnostic yield of Type B2 vessels in evaluating tumor invasion depth. Studies have found that as the size of the area where Type B2 vessels were observed increased, the pathological tumor invasion depth tended to increase [7, 8]. Consequently, when the observed area was small, the probability of pEP/LPM was high; when the observed area was large, the probability of pSM2-3 was higher. The cut-off values for the tumor invasion depth were 4 mm for pEP/LPM and pMM/SM1 and 10 mm for pMM/SM1 and pSM2-3. In addition, the macroscopic type of the Type B2 vessel area was associated with submucosal invasion. When an elevation (macroscopic type, 0-I) or deep depression (macroscopic type, 0-III) is observed within the B2 vessel area, there is a tendency for pSM2-3 [8]. However, the effectiveness of these additional findings, such as the size of the B2 vessel area and macroscopic type 0-I/0-III in the B2 vessel area, is yet to be confirmed.

This prospective study aims to verify the usefulness of considering the size and macroscopic type of Type B2 vessel area and depth evaluation based on the JES magnifying endoscopy classification in cMM/SM1 cancers.

## Methods

### Study design

**Fig. 1:**
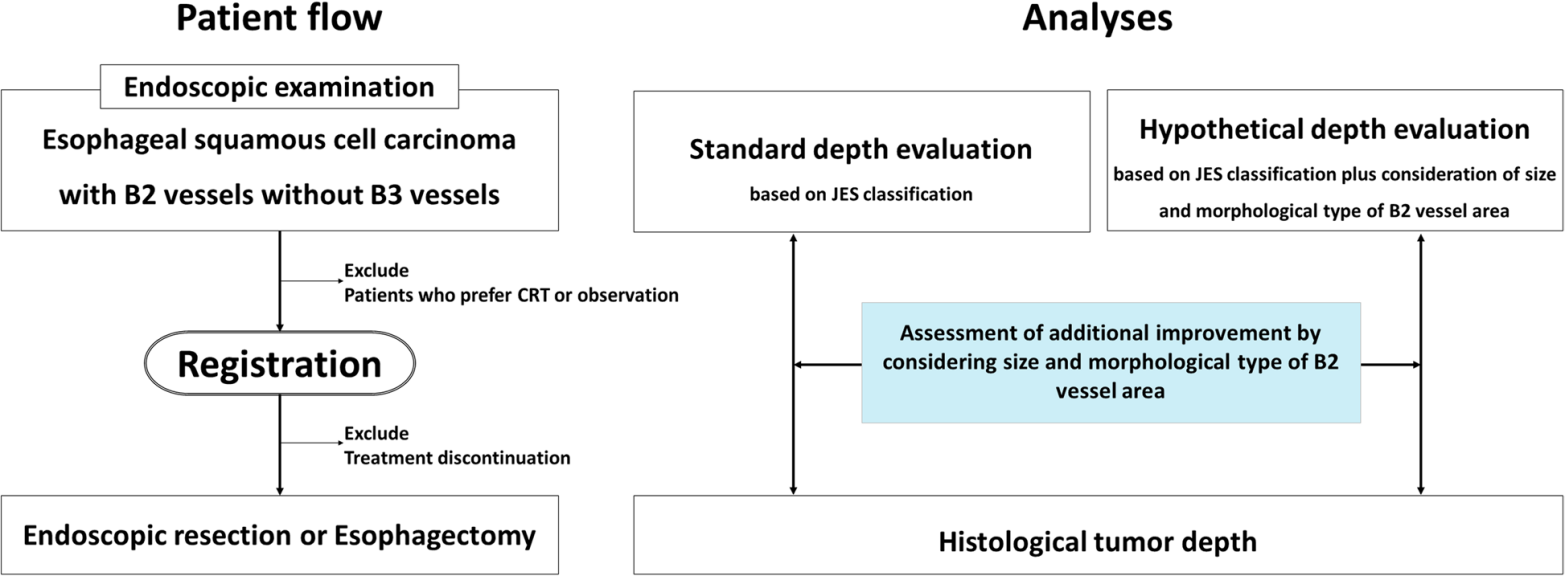
Patient flow and analyses. CRT, chemoradiotherapy; JES, Japan Esophageal Society

This is a multicenter prospective observational study. Endoscopic examinations will be performed in 13 hospitals in Japan. Tumor invasion depth will be evaluated based on only the JES classification (standard-depth evaluation) and the JES classification with additional findings (hypothetical-depth evaluation) for the same patients (Fig. 1). The study protocol will be performed following the Declaration of Helsinki and is approved by the Ethical Review Board of Shizuoka Cancer Center (IRB approval number: T2022–53–2022-1–2). The trial is registered in the Clinical Trials Registry of the University Hospital Medical Information Network (UMIN-CTR) as UMIN000051145. The date of the first registration was 23/5/2023.

### Study population

Patients who meet all the following inclusion criteria and do not meet any exclusion criteria will be eligible for registration.

#### Inclusion criteria

1. Diagnosis of histologically confirmed squamous cell carcinoma or clinically diagnosed esophageal squamous cell carcinoma.
2. Clinical T1 esophageal cancer where Type B2 vessels will be observed but Type B3 vessels will not.
3. The part where Type B2 vessels are observed will be considered the tumor’s deepest part.
4. The main sites of the tumor are the cervical, thoracic, and abdominal esophagi [9].
5. The maximum tumor diameter is ≤ 50 mm.
6. Undergoing endoscopic or surgical resection as initial treatment. However, only patients without planned preoperative chemotherapy for overlapping head, neck, or gastric cancer are eligible.
7. No simultaneous esophageal advanced cancer.
8. No history of surgical esophagectomy.
9. No history of radiation therapy to the esophagus, lung field, mediastinum, or treatment for other cancer types.
10. Previous endoscopic resection for esophageal cancer is not a concern. However, the patient is ineligible if the lesion is within 10 mm of the scar from the previous treatment.
11. The age at registration is ≥ 18 years.
12. Performance status will be assessed following the ECOG criteria and should range between 0 and 2. [10]
13. The patient agrees to provide written consent for participation in the trial.

#### Exclusion criteria

1. Systemic infections requiring treatment.
2. Women who are pregnant, who may be pregnant, or who are breastfeeding.
3. Psychiatric disorders, psychiatric symptoms, or cognitive impairments that make participation in the trial difficult.
4. Other patients who are deemed inappropriate by the registering physician.

### Tumor invasion depth evaluation

#### Standard depth evaluation

Tumor invasion depth will be diagnosed based on the JES classification (Table 1). Specifically, Type B2 vessels are defined as abnormal microvessels with severe irregularity or highly dilated abnormal vessels without loop-like formation." Since previous studies have shown that interobserver and intraobserver agreements for the JES classification were sufficient [11, 12], a central judgment of the JES classification is considered unnecessary in this study. However, typical endoscopic images of Type B2 vessels were confirmed before the study. Type B2 vessel is evaluated where ulcer or erosion is not observed to avoid the effect of B2-mimicking vessels that appear by inflammation [13].

#### Hypothetical depth evaluation

Before initiating the study, we verified the sizes of biopsy forceps available in the market and gained an understanding of the forceps sizes used at each facility. Based on this information, the area of the type B2 vessel will be measured in millimeters using the forceps size as a guide. Measurements < 1 mm will be rounded to 1 mm. If Type B2 vessels are present in multiple areas, then the largest area will be recorded. Macroscopic types are defined following the Treatment Guidelines for Esophageal Cancer [14, 15]. Namely, 0-I is defined as a protruding lesion that is ≥ 1 mm in height, and 0-III is a depressed lesion that is estimated to reach the muscularis mucosa or deeper.

The criteria for judging tumor invasion depth are discussed multiple times by the JES superficial esophageal cancer depth diagnosis criteria study group, and a consensus is reached (Table 2). The anticipated high diagnostic concordance using biopsy forceps as a reference led to the omission of central evaluation in this study.

1. A Lesion with a Type B2 vessel area of ≤ 4 mm is diagnosed as cEP/LPM.
2. A lesion with a Type B2 vessel area of ≥ 10 mm is diagnosed as cSM2-3.
3. I and/or 0-III macroscopic types of the Type B2 vessel area are diagnosed as cSM2-3.
4. When conflicting findings of a B2 vessel area of ≤ 4 mm and 0-I/0-III or Type B2 vessel area are observed in one Type B2 vessel area, the lesion is diagnosed as cSM2-3.
5. When multiple Type B2 vessel areas are observed, the depth is determined based on the deepest finding. Specifically, the order of adoption is 0-I/0-III of Type B2 vessel area, the size of B2 vessel area ≥ 10 mm, the size of Type B2 vessel area between 4 and 10 mm, and the size of Type B2 vessel area ≤ 4 mm.

**Table 2:** Criteria for hypothetical depth diagnosis

	Corresponding tumor invasion depth
B2 vessel area ≤ 4 mm	EP, LPM
B2 vessel area between 4–10 mm without additional findings^a^	MM, SM1
B2 vessel area ≥ 10 mm or macroscopic type 0-I/0-III in B2 vessel area	SM2-3

*MM* Muscularis mucosa, *SM1* Upper third of the submucosal layer, *EP/LPM* Epithelial/lamina propria

^a^Additional findings are B2 vessel area ≤ 4 mm, B2 vessel area ≥ 10 mm, and macroscopic type 0-I/0-III in B2 vessel area

### Treatment

Endoscopic resection or esophagectomy should be performed within 12 weeks of upper gastrointestinal endoscopy to minimize the effect of treatment delay on tumor invasion depth. It is considered a protocol deviation if the duration is > 12 weeks.

### Histological evaluation

After endoscopic resection, the specimens will be dissected at 2–3 mm intervals, and the histological tumor invasion depth will be determined. In surgical cases, the specimens will be dissected at intervals of 3 mm whenever possible. Histological diagnoses will be made based on the Treatment Guidelines for Esophageal Cancer [14, 15]. The tumor invasion depth is defined as follows: pEP, cancer remains within the epithelium; pLPM, cancer remains within the lamina propria; pMM, cancer invades the muscularis mucosa; pSM1, cancer remains within 200 μm of the submucosa from the muscularis mucosa; and pSM2, cancer invades beyond 200 μm into the submucosa. Desmin immunostaining is recommended for evaluating the muscularis mucosae.

Esophageal squamous cell carcinoma is a disease encountered daily and is not difficult to diagnose. The handling of specimens is also described in detail in the esophageal cancer handling guidelines; therefore, it is assumed that there is a slight variation in diagnosis between facilities. Therefore, in this study, pathological diagnosis is used to determine the histological depth of esophageal cancer, and no central judgment is performed.

### Registration and data collection

After obtaining written consent from the participants, the trial physicians will register the case on the web built into the UMIN Internet data and information system for clinical and epidemiological research, cloud version (UMIN INDICE cloud, https://www.umin.ac.jp/indice/cloud.html). Participants will be assigned a deidentified number. Data will be collected and stored in the UMIN INDICE cloud using an electronic case record form.

The following data will be collected at the time of registration in a prospective manner: duration of endoscopic observation, model of endoscope used, tumor location, tumor size, macroscopic type, number of areas where type B2 vessels are observed, size of type B2 vessel areas, and macroscopic type of type B2 vessel areas. The following data will be collected after registration: treatment method, en bloc or piecemeal resection, histological type, histological tumor size, size of the resected specimen, histological tumor invasion depth, lymphatic invasion, vascular invasion, resection margin, and presence of lymph node metastasis.

### Endpoints

#### Primary endpoint

This study’s primary endpoint is accuracy, defined as the proportion of cases in which the preoperative depth diagnosis matches the histological depth diagnosis after resection.

#### Secondary endpoints

The secondary endpoints include the following: the proportion of pSM2-3 when the area in which B2 vessels are observed is > 10 mm, the proportion of pSM2-3 when the macroscopic type of the observed B2 vessel area is 0-I or 0-III, the proportion of pSM2-3 when the area in which B2 vessels are observed is > 10 mm or the macroscopic type is 0-I or 0-III, the proportion of pEP/LPM when the area in which B2 vessels are observed is < 4 mm, PPV, sensitivity, specificity, likelihood ratio, a relation between the size of the B2 vessel area and the histological tumor invasion depth, a relation between the number of B2 vessel areas and the histological tumor invasion depth, and the relationship between the macroscopic type of the B2 vessel area and histological tumor invasion.

Furthermore, if the proportion of pSM2-3 when the area in which B2 vessels are observed is > 10 mm, the proportion of pSM2-3 when the macroscopic type of the observed B2 vessel area is 0-I or 0-III, and the proportion of pEP/LPM when the area in which B2 vessels are observed is < 4 mm exceeds 50%, these findings are considered to have an impact on the clinical diagnosis and are therefore included as secondary endpoints.

### Sample size calculation

Following a review of retrospective studies, the PPV for diagnosing cMM/SM1 using the JES classification was 55.7% [3]. In addition, a recent prospective study showed that the PPV of cMM/SM1 was 47% [13]. Because the PPV for cMM/SM1 based on the standard-depth evaluation is calculated using the same formula as that used for the accuracy, the accuracy for cMM/SM1 based on this study’s standard-depth evaluation is set at 50%. Because the accuracy was 77.7% in a previous study when considering the size of the B2 vessel area [13], it is assumed that considering the size and morphological type of the B2 vessel area would result in an improved accuracy of at least 15% in this study. The research group agreed that a 15% improvement in accuracy is clinically meaningful for improving the preoperative evaluation of tumor invasion depth. Therefore, assuming that the accuracy for the hypothetical-depth assessment is 65%, which is obtained by adding a 15% improvement to the accuracy of the standard-depth evaluation of 50%, when considering the size and morphological type of the B2 vessel area, the required lesion number is determined using an accurate test for the binomial distribution with a two-sided alpha error of 0.05, power of 90%, and the required lesion number of 121 lesions. After considering a dropout rate of approximately 10%, the necessary lesion number is calculated as 135.

### Planned statistical analyses

The main analysis for the primary endpoint, which is performed after all data associated with the endpoint are fixed after registration, aims to verify whether the accuracy can be improved by considering the size and macroscopic type of the B2 vessel area in addition to the standard-depth evaluation based on the JES classification. Suppose the diagnostic accuracy of the standard-depth assessment is statistically improved compared with that of the standard-depth evaluation. In that case, we will conclude that the hypothetical-depth review is a more useful diagnostic method. Suppose the accuracy of the hypothetical-depth evaluation does not significantly exceed that of the standard-depth evaluation. In that case, it can be concluded that standard-depth evaluation remains a useful diagnostic method. The overall two-sided significance level is set at 5%.

#### Analysis of the primary endpoint

The calculation formula for the standard-depth evaluation is: (lesions diagnosed as cMM/SM1 and pMM/SM1 [B]) / (total lesions [A + B + C]) (Table 3), and for the depth evaluation, [(lesions diagnosed as cEP/LPM and pEP/LPM [a]) + ((lesions diagnosed as cMM/SM1 and pMM/SM1 [e]) + (lesions diagnosed as cSM2-3 and pSM2-3 [i]))] / (total lesions [a + b + c + d + e + f + g + h + i]) (Table 4). A point estimate is calculated for accuracy, and a 95% confidence interval is calculated based on the exact binomial distribution. The corresponding *p*-value is also calculated.

**Table 3:** Relationship between standard depth evaluation and histological depth diagnosis

	pEP/LPM	pMM/SM1	pSM2-3
cMM/SM1 (B2 vessel)	A	B	C

$$\begin{array}{l}\mathrm{Accuracy}\;\mathrm{of}\;\mathrm{standard}-\mathrm{depth}\;\mathrm{evaluation}\;(\%)\\=(\mathrm{number}\;\mathrm{of}\;\mathrm{lesions}\;\;\mathrm{diagnosed}\;\mathrm{as}\;\mathrm{cMM}/\text{SM}1\;\mathrm{and}\;\mathrm{confirmed}\;\mathrm{as}\;\mathrm{pMM}/\text{SM}1)/(\mathrm{total}\;\mathrm{number}\;\mathrm{of}\;\mathrm{lesions})\times100=\lbrack\text{B}\rbrack/\lbrack\text{A}+\text{B}+\text{C}\rbrack\text{x}100\end{array}$$

*MM* Muscularis mucosa, *SM1* Upper third of the submucosal layer, *EP/LPM* Epithelial/lamina propria

**Table 4:** Relationship between hypothetical depth evaluation and histological depth diagnosis

	pEP/LPM	pMM/SM1	pSM2-3
cEP/LPM (B2 vessel area ≤ 4 mm)	a	b	c
cMM/SM1 (B2 vessel area between 4–10 mm without additional findings^a^)	d	e	f
cSM2-3 (B2 vessel area ≥ 10 mm or macroscopic type 0-I/0-III in B2 vessel area)	g	h	i

^a^Additional findings: B2 vessel area ≤ 4 mm, B2 vessel area ≥ 10 mm, macroscopic type 0-I/0-III in B2 vessel area

$$\begin{array}{l}\mathrm{Accuracy}\;\mathrm{of}\;\mathrm{hypothetical}-\mathrm{depth}\;\mathrm{evaluation}\;(\%)\\=(\lbrack\mathrm{number}\;\mathrm{of}\;\mathrm{lesions}\;\mathrm{diagnosed}\;\mathrm{as}\;\mathrm{cEP}/\mathrm{LPM}\;\mathrm{and}\;\mathrm{confirmed}\;\mathrm{as}\;\mathrm{pEP}/\text{LPM})+(\mathrm{number}\;\mathrm{of}\;\mathrm{lesions}\;\mathrm{diagnosed}\;\mathrm{as}\;\mathrm{cMM}/\text{SM}1\;\mathrm{and}\;\mathrm{confirmed}\;\mathrm{as}\;\mathrm{pMM}/\text{SM}1)+(\mathrm{number}\;\mathrm{of}\;\mathrm{lesions}\;\mathrm{diagnosed}\;\mathrm{as}\;\mathrm{cSM}2\;\mathrm{or}\;\mathrm{deeper}\;\mathrm{and}\;\mathrm{confirmed}\;\mathrm{as}\;\mathrm{pSM}2\;\mathrm{or}\;\mathrm{deeper}\rbrack)/(\mathrm{total}\;\mathrm{number}\;\mathrm{of}\;\mathrm{lesions})\times100.\\=(\text{a}+\text{e}+\text{i})/(\text{a}+\text{b}+\text{c}+\text{d}+\text{e}+\text{f}+\text{g}+\text{h}+\text{i})\mathrm x100\end{array}$$

*MM* muscularis mucosa, *SM1* upper third of the submucosal layer, *EP/LPM* epithelial/lamina propria

#### Analysis of proportion

A confidence interval is calculated based on the exact binomial distribution.

#### Analysis of PPV, sensitivity, and specificity

For cases where the histological tumor invasion depth is obtained after endoscopic resection or esophagectomy, the PPV, sensitivity, and specificity will be calculated as described above, and *p*-values will be calculated using Fisher's exact test, if necessary.

The PPV of the standard-depth evaluation is calculated for cMM/SM1 as follows:

1. cMM/SM1
  $$\mathrm{PPV}(\%)=(\mathrm{number}\;\mathrm{of}\;\mathrm{lesions}\;\mathrm{diagnosed}\;\mathrm{as}\;\mathrm{cMM}/\text{SM}1\;\mathrm{and}\;\mathrm{positive}\;\mathrm{for}\;\mathrm{pMM}/\text{SM}1)/(\mathrm{total}\;\mathrm{number}\;\mathrm{of}\;\mathrm{lesions}\;\mathrm{diagnosed}\;\mathrm{as}\;\mathrm{cMM}/\text{SM}1)\times100=\text{B}/(\text{A}+\text{B}+\text{C})\times100$$

The PPV in the hypothetical-depth evaluation will be calculated for cEP/LPM, cMM/SM1, and cSM2-3 as follows:

1. cEP/LPM
  $$\mathrm{PPV}\;(\%)=(\mathrm{number}\;\mathrm{of}\;\mathrm{lesions}\;\mathrm{diagnosed}\;\mathrm{as}\;\mathrm{cEP}/\mathrm{LPM}\;\mathrm{and}\;\mathrm{positive}\;\mathrm{for}\;\mathrm{pEP}/\text{LPM})/(\mathrm{total}\;\mathrm{number}\;\mathrm{of}\;\mathrm{lesions}\;\mathrm{diagnosed}\;\mathrm{as}\;\mathrm{cEP}/\text{LPM})\times100=\text{a}/(\text{a}+\text{b}+\text{c})$$
2. cMM/SM1
  $$\text{PPV}\;(\%)=(\mathrm{number}\;\mathrm{of}\;\mathrm{lesions}\;\mathrm{diagnosed}\;\mathrm{as}\;\mathrm{cMM}/\text{SM}1\;\mathrm{and}\;\mathrm{positive}\;\mathrm{for}\;\mathrm{pMM}/\text{SM}1)/(\mathrm{total}\;\mathrm{number}\;\mathrm{of}\;\mathrm{lesions}\;\mathrm{diagnosed}\;\mathrm{as}\;\mathrm{cMM}/\text{SM}1)\times100=\text{d}/(\text{d}+\text{e}+\text{f}).$$
3. cSM2-3
  $$\text{PPV}(\%)=(\mathrm{number}\;\mathrm{of}\;\mathrm{lesions}\;\mathrm{diagnosed}\;\mathrm{as}\;\mathrm{cSM}2-3\;\mathrm{and}\;\mathrm{pSM}2-3)/(\mathrm{total}\;\mathrm{number}\;\mathrm{of}\;\mathrm{lesions}\;\mathrm{diagnosed}\;\mathrm{as}\;\mathrm{cSM}2-3)\times100=\text{i}/(\text{g}+\text{h}+\text{i})$$

The sensitivity and specificity of cEP/LPM, cMM/SM1, and cSM2-3 will be calculated for depth evaluation as follows:

1. cEP/LPM
  $$\mathrm{Sensitivity}\;(\%)=(\mathrm{number}\;\mathrm{of}\;\mathrm{lesions}\;\mathrm{diagnosed}\;\mathrm{as}\;\mathrm{cEP}/\mathrm{LPM}\;\mathrm{and}\;\mathrm{confirmed}\;\mathrm{as}\;\mathrm{pEP}/\text{LPM})/(\mathrm{number}\;\mathrm{of}\;\mathrm{lesions}\;\mathrm{confirmed}\;\mathrm{as}\;\mathrm{pEP}/\text{LPM})\times100=\text{a}/(\text{a}+\text{d}+\text{g})\times100$$
  $$\text{Specificity}(\%)=(\mathrm{number}\;\mathrm{of}\;\mathrm{lesions}\;\mathrm{not}\;\mathrm{diagnosed}\;\mathrm{as}\;\mathrm{cEP}/\mathrm{LPM}\;\mathrm{and}\;\mathrm{confirmed}\;\mathrm{as}\;\mathrm{pEP}/\text{LPM})/(\mathrm{number}\;\mathrm{of}\;\mathrm{lesions}\;\mathrm{not}\;\mathrm{confirmed}\;\mathrm{as}\;\mathrm{pEP}/\text{LPM})\times100=(\text{e}+\text{h}+\text{f}+\text{i})/(\text{b}+\text{e}+\text{h}+\text{c}+\text{f}+\text{i})\times100$$
2. cMM/SM1
  $$\mathrm{Sensitivity}(\%)=(\mathrm{number}\;\mathrm{of}\;\mathrm{lesions}\;\mathrm{diagnosed}\;\mathrm{as}\;\mathrm{cMM}/\text{SM }1\;\mathrm{and}\;\mathrm{confirmed}\;\mathrm{as}\;\mathrm{pMM}/\text{SM}1)/(\mathrm{number}\;\mathrm{of}\;\mathrm{lesions}\;\mathrm{confirmed}\;\mathrm{as}\;\mathrm{pMM}/\text{SM}1)\times100=\text{e}/(\text{b}+\text{e}+\text{h})\times100$$
  $$\text{Specificity }(\%)=(\mathrm{number}\;\mathrm{of}\;\mathrm{lesions}\;\mathrm{not}\;\mathrm{diagnosed}\;\mathrm{as}\;\mathrm{cMM}/\text{SM}1\;\mathrm{and}\;\mathrm{confirmed}\;\mathrm{as}\;\mathrm{pMM}/\text{SM}1)/(\mathrm{number}\;\mathrm{of}\;\mathrm{lesions}\;\mathrm{not}\;\mathrm{confirmed}\;\mathrm{as}\;\mathrm{pMM}/\text{SM}1)\times100=(\text{a}+\text{g}+\text{c}+\text{i})/(\text{a}+\text{d}+\text{g}+\text{c}+\text{f}+\text{i})\times100$$
3. cSM2-3
  $$\text{Sensitivity}(\%)=(\mathrm{number}\;\mathrm{of}\;\mathrm{lesions}\;\mathrm{diagnosed}\;\mathrm{as}\;\mathrm{cSM}2-3\;\mathrm{and}\;\mathrm{confirmed}\;\mathrm{as}\;\mathrm{pSM}2-3)/(\mathrm{number}\;\mathrm{of}\;\mathrm{lesions}\;\mathrm{confirmed}\;\mathrm{as}\;\mathrm{pSM}2-3)\times100=\text{i}/(\text{c}+\text{f}+\text{i})\times100$$
  $$\text{Specificity }(\%)=(\mathrm{number}\;\mathrm{of}\;\mathrm{lesions}\;\mathrm{not}\;\mathrm{diagnosed}\;\mathrm{as}\;\mathrm{cSM}2-3\;\mathrm{and}\;\mathrm{confirmed}\;\mathrm{as}\;\mathrm{pSM}2-3)/(\mathrm{number}\;\mathrm{of}\;\mathrm{lesions}\;\mathrm{not}\;\mathrm{confirmed}\;\mathrm{as}\;\mathrm{pSM}2-3)\times100=(\text{a}+\text{d}+\text{b}+\text{e})/(\text{a}+\text{d}+\text{g}+\text{b}+\text{e}+\text{h})\times100$$

#### Analysis of likelihood ratios

For cases in which the histological tumor invasion depth is obtained after endoscopic resection or esophagectomy, the likelihood ratios will be calculated as sensitivity / (1-specificity) for each additional finding, such as B2 vessel area ≤ 4 mm, B2 vessel area ≥ 10 mm, and macroscopic type 0-I/0-III in the B2 vessel area.

#### Relation between size of B2 vessel area and histological tumor invasion depth

For cases in which the histological tumor invasion depth is obtained after endoscopic resection or esophagectomy, a paired-samples t-test will be performed to reject the null hypothesis test, which is whether the two groups of pEP/LPM and pMM/SM1 are equal in size of the observed B2 vessel area. The pMM/SM1 and pSM2-3 groups will use the same test. The cut-off value will be calculated based on the receiver operating characteristic (ROC) curve.

#### Relation between the number of observed B2 blood vessel areas and histological depth

For lesions in which a histological depth diagnosis is obtained through endoscopic resection or esophagectomy, we will also examine the number of observed B2 blood vessel areas (single location vs. multiple locations) between the two groups: pEP/LPM and pMM/SM1 and pMM/SM1 and pSM2-3. Fisher's exact test using p-values for contingency tables will be used for intergroup comparisons.

#### *Relation between* macroscopic type of B2 vessel area* and histological tumor invasion depth*

We will focus on macroscopic types 0-I and 0-III of the observed B2 vascular areas and calculate *p*-values using Fisher's exact test for tables concerning pMM/SM1 and pSM2-3, respectively.

## Discussion

Tumor invasion depth significantly impacts the selection of treatment for superficial esophageal cancer. Diagnosing preoperative tumor invasion depth is paramount because physical invasiveness significantly differs among treatments. However, the diagnostic yield of the JES classification for preoperative depth diagnosis is insufficient, particularly for cMM/SM1 cancer. A multicenter prospective study evaluating the diagnostic value of endoscopic ultrasonography (EUS) after magnifying endoscopy showed that adding EUS did not improve the accuracy of the tumor invasion depth diagnosis [16, 17]. This prospective study can establish a new approach for preoperative tumor invasion depth diagnosis based on high-level evidence, ultimately optimizing the process of diagnosing cT1 esophageal cancer.

Improving the PPV of cMM/SM1 is a pressing concern in the JES magnifying endoscopic classification. However, this study designated the PPV as a secondary endpoint because special attention is required when evaluating its improvement. There are specific patterns in which the overall diagnostic performance may deteriorate even if the PPV shows improvement. For instance, the PPV may increase when categorizing low-confidence cases as cEP/LPM or cSM2-3 instead of cMM/SM1, but the overall diagnostic performance may decrease. Therefore, we set accuracy as the primary endpoint for monitoring diagnostic performance. Nevertheless, even if the primary endpoint shows significant improvement, the clinical significance of considering the size and macroscopic type of the B2 vessel area is limited if the PPVs are substantially lower in the hypothetical-depth evaluation than in the standard-depth evaluation. Therefore, we will interpret the study results after confirming a higher percentage of PPVs for cMM/SM1 through the hypothetical-depth review compared with the standard-depth evaluation.

Suppose the effectiveness of considering the size and macroscopic type of the B2 vessel area in the accuracy of tumor invasion depth diagnosis is verified. In that case, the current JES classification can be further subdivided, enabling the selection of more appropriate treatments for patients with superficial esophageal cancer. However, the expected clinical effect cannot be achieved if the effectiveness is not confirmed. Therefore, candidate findings can still be identified, and exploratory analyses can be conducted to examine the relationship between the size of the B2 vessel area and tumor invasion depth. In addition, the optimal cut-off value reflecting tumor invasion depth can be reevaluated using data from this prospective study in future verification trials.

This study design has several limitations. First, this prospective study has an observational design. The effectiveness of the additional findings can be confirmed; however, their clinical effects cannot be fully elucidated in this study. For instance, the extent to which treatment modalities have been altered based on the insights gained in this study and whether there are differences in subsequent outcomes remain unclear. To demonstrate this, a randomized controlled trial (RCT) with two groups–one using standard-depth evaluation and the other using hypothetical-depth evaluation–is necessary. However, conducting an RCT is not feasible due to the physically and mentally invasive nature of such a trial, which would be unacceptable as regards ethical standards. Second, we will only include patients with superficial esophageal cancer diagnosed with cMM/SM1 based on the B2 vessel. Ideally, when evaluating the comprehensive diagnostic performance, cases of cEP/LMP and cSM2-3 should also be included. However, most cases in our dataset are cEP/LPM, and the PPV of cEP/LPM has been shown as excellent in previous studies. Given the limited resources available for this study, we focus on cMM/SM1 cases.

Collecting reliable clinical data through the Japan BEES study may provide interesting insights into the JES magnifying endoscopic classification, specifically the B2 vessel category. These insights could be valuable for clinical decision-making and may facilitate the adoption of more appropriate evidence-based medical practices. Third, in actual clinical practice, comprehensive judgments involving exploratory submucosal injection, EUS, computed tomography, and other modalities are likely used to determine tumor depth. Therefore, caution is needed in extrapolating clinical effectiveness based solely on the results of this study.

## Trial status

Current protocol, version 1.2, issue date 6.5.2023. Recruitment started on 16.6.2023 and is planned to be completed on 5.2025.

## Data Availability

The datasets used and/or analyzed in the current study are available from the corresponding author, MY, upon reasonable request.

## Abbreviations

ER: Endoscopic resection
cT1a: Clinical T1a
CRT: Chemoradiotherapy
cT1b: Clinical T1b
OS: Overall survival
pT1a: Pathological T1a
pT1b: Pathological T1b
EP/LPM: Epithelial/lamina propria
MM: Muscularis mucosa
SM: Submucosal invasive cancer
JES: Japan Esophageal Society
PPV: Positive predictive value
UMIN-CTR: Clinical Trials Registry of University Hospital Medical Information Network
ECOG: Eastern Cooperative Oncology Group
